# From Waste to Technological Products: Bioplastics Production from Proteins Extracted from the Black Soldier Fly

**DOI:** 10.3390/polym17111582

**Published:** 2025-06-05

**Authors:** Alessia Di Pasquale, Marina Zoccola, Ashish Mohod, Giulia Dalla Fontana, Anastasia Anceschi, Sara Dalle Vacche

**Affiliations:** 1 Department of Applied Science and Technology, Politecnico di Torino, Corso Duca degli Abruzzi 24, 10129 Torino, Italy; aledipa9@gmail.com (A.D.P.); sara.dallevacche@polito.it (S.D.V.); 2 CNR-STIIMA, Italian National Research Council, Institute of Intelligent Industrial Technologies and Systems for Advanced Manufacturing, Corso G. Pella 16, 13900 Biella, Italy; ashish.mohod@cnr.it (A.M.); giulia.dallafontana@cnr.it (G.D.F.); anastasia.anceschi@stiima.cnr.it (A.A.)

**Keywords:** bioplastics, Black Soldier Fly, proteins, polyvinyl alcohol, biofactory, high-value products

## Abstract

The need to find sustainable solutions to conventional plastics has driven research into alternative materials, including bioplastics, which represent a promising option for reducing pollution and enhancing the value of renewable resources. In this study, bioplastics made from polyvinyl alcohol (PVA) and proteins extracted from the larvae of Black Soldier Fly (BSF), an insect capable of converting organic waste into high-value biomass, were produced and characterized. The proteins were obtained by hydrolysis of defatted BSF larvae with superheated water, avoiding harsh chemical reagents. Next, polymer films were fabricated by mixing PVA and hydrolyzed BSF proteins in different proportions and analyzed for morphological, physical-chemical, mechanical and biodegradability characteristics. The results obtained show that as the BSF protein content increases, the films show a reduction in thermal stability and mechanical properties, and also, they exhibit higher biodegradability, correlated with higher wettability, solubility and ability to absorb moisture. This research highlights the value of using organic waste-fed insects as a resource for bioplastic production, offering an alternative to traditional polymers and contributing to the transition to sustainable materials.

## 1. Introduction

*Hermetia illucens*, also known as the Black Soldier Fly, is an insect of the order Diptera and the family Stratiomyidae. This species is native to South America, but is currently widespread on a cosmopolitan level, due to its ability to adapt to many different environments and food sources [1]. The life cycle of BSF consists of four main stages, with the first stage being eggs laid near decomposing organic material. The larvae then come out of the eggs and grow rapidly, accumulating body mass. When they reach maturity, they become first prepupae, where they stop feeding and their skin hardens, and immediately after pupae, where they undergo the metamorphosis into winged adults, which concentrate exclusively on reproduction [2]. BSF larvae have the potential to bioconvert a large variety of organic substrates in their body mass, and they can be used in waste management, requiring a relatively low amount of water, land and energy to be reared [3,4,5].

As insects are ‘farmed animals’ [6], the EU legislation does not allow us to feed them with organic waste [7]. However, to implement the circularity level of the EU insect sector and help it to reach its full potential, the International Platform of Insects for Food and Feed [8] has identified as a key research priority the diversification of the allowed growing substrates. Moreover, around the world, such legislation does not apply and BSF larvae are a very valuable tool for waste management [9,10,11,12,13]. This process has several advantages compared to traditional organic waste treatment strategies. It could make a very meaningful contribution to meeting several of the UN Sustainable Development Goals (SDGs) for 2030 and thereafter.

The present work focused on extracting proteins from BSF larvae raised on food scraps and their use for technological purposes for bioplastic production.

The European Union generated 59.2 million tons of food waste in 2022, corresponding to around 132 kg per inhabitant [14]. The recycling of food waste to produce commercial products and energy is fast picking up in the scientific community as a sustainable option [15]. Food waste is characterized by a variable chemical composition depending on its origin of production, while feeding insects with food waste allows for concentrating the waste in their body mass, which can be biorefined, obtaining fat, protein and chitin [16].

After the grease extraction, proteins can be extracted from the insect body mass using strong bases, in particular NaOH, which is the most widespread and widely established method [17,18], or enzymes [1,19].

In this study, to extract proteins from BSF larvae, superheated water at a temperature of 160 °C corresponding to a pressure of 4.5 bar was used, which offers the advantages of being an environmentally sustainable process, not requiring complex and expensive purification steps, as well as sterilizing the material and ensuring a safe protein end product.

Superheated water is defined as liquid water under pressure at a temperature between 100 °C (boiling temperature at atmospheric pressure) and 374 °C (critical water temperature) [20]. As far as the protein is concerned, superheated water has been used to obtain keratins from wool and feathers [21,22] and to extract protein from insects to obtain natural dyes [23] or to remove proteins and isolate chitin from BSF exuviae [24].

In this study, the proteins extracted from BSF with superheated water were blended with PVA for bioplastic production to overcome the disadvantages of the protein’s fragile structure and poor mechanical properties [25]. Other authors had already used the protein extracted from BSF prepupae to produce bioplastics in a mixture with glycerol [26] or glycerol as a plasticizer and citric acid as a cross-linking agent [27]. In our work, PVA was chosen because it is a hydrophilic polymer, water soluble, with a good film-forming ability [28] and compatible with proteins in film production [29,30]. Moreover, PVA shows high mechanical properties and good chemical resistance but poor thermal properties, although its properties depend largely on the molecular weight and degree of hydrolysis. PVA is not considered a biodegradable polymer in all environments, as its biodegradability depends on the microorganisms present, and environmental conditions of temperature, pH and humidity [31].

The bioplastic production from insect larvae feeding on food waste reduces dependence on polymers from fossil sources. The most widely used polymers are low-density polyethylene (LDPE), high-density polyethylene (HDPE), polyvinyl chloride, polystyrene and polypropylene and polyethylene terephthalate (PET). A major problem related to their high durability is the accumulation in soil and water [32]. In addition, the synthetic polymers are derived from non-renewable sources, particularly based on petroleum resources. Moreover, there are some problems related to their recycling; For example, fusion-based recycling to produce new polymeric material can lead to significant deterioration of the material obtained due to the presence of moisture content and residual catalysis in treated waste polymer [33].

Instead, biopolymers are characterized by their derivation from renewable resources or because they are biodegradable, which means they can degrade in natural environments or under specific industrial conditions [34,35]. In the case of insect proteins, both requirements are met.

Biodegradable polymers are transformed into water, carbon dioxide, methane or biomass through biological processes, making them particularly useful for applications such as single-use materials where end-of-life management is challenging [36]. Bioplastics may be produced synthetically as polylactic acid (PLA) or as natural biopolymers such as cellulose and its derivatives, starch, proteins or mixtures thereof [37]. The mechanical characteristics of biopolymers are widely variable from poly(glycolic acid) (PGA), which has a high tensile strength of 60 MPa, to starch, which has a tensile strength of 5 MPa. Often, tensile strength and elongation are inversely related, and generally, biopolymers have lower mechanical properties than synthetic polymers [38]. The biodegradability of bioplastics is difficult to quantify as it depends not only on the type of polymer but also on the additives used in its formulation, which increase the durability of the material. Additionally, of course, it depends on the characteristics of the environment in which biodegradability is tested [39]. The bioplastics produced in this study could find application as packaging materials or in agriculture, where traditional plastics, such as low-density polyethylene (LDPE), are widely used as mulching films. Although appreciated for their low cost and good mechanical and optical performance, during the degradation process, they reduce into fragments that have to be removed [40].

On the other hand, bioplastics from proteins are designed not only to degrade completely without leaving harmful residues but also to release carbon and organic nitrogen that fertilize the soil and support crop growth [26,41,42]. In this study, bioplastics were obtained from BSF larvae proteins extracted by a green method using only water under pressure and mixed with PVA in different proportions using water as a common solvent. The bioplastics were characterized via chemical (spectroscopy in the medium infrared—FTIR) thermal, mechanical, and morphological analyses, and parameters such as water solubility, moisture absorption, contact angle, and biodegradability in soil were measured, aiming to characterize the different films and direct them towards specific uses.

## 2. Materials and Methods

### 2.1. Materials

The proteins were extracted from BSF larvae reared at the experimental center of the Department of Zootechnical Sciences of the University of Turin in Carmagnola (Turin), in a climate chamber with controlled temperature and humidity (29 ± 0.5 °C, relative humidity 60 ± 5%) and constant ventilation. PVA was supplied by the company F.lli Citterio S.p.A. (Monza-Italy—average m.w. 100,000) in filament form. Distilled water was prepared using the Millipore apparatus. Hexane, petroleum ether and hydrochloric acid were of analytical grade and were purchased from Merck (Milano—Italy).

### 2.2. Methods

#### 2.2.1. BSF Larvae and PVA Preparation

**Figure 1:**
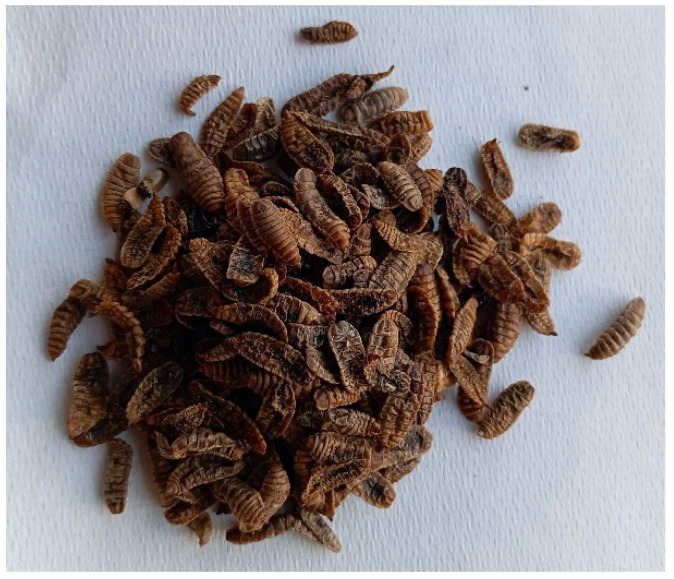
Dried BSF larvae.

BSF larvae at the last larval age were dried (see Figure 1), washed 3 times in deionized water, further dried at 55 °C for 24 h and finely ground in a blender.

The larval powder was then extracted in a Soxhlet apparatus for 4 h using hexane as a solvent to remove larval fat. From the defatted larvae, the proteins were extracted. The PVA yarn was extracted in a Soxhlet apparatus for 4 h using petroleum ether as a solvent to remove fats and finishing products.

#### 2.2.2. Protein Extraction from BSF Larvae and Their Characterization

Superheated water was used for protein extraction in a laboratory reactor (Amar Equipment, Mumbai, India, model number 1-T-A-CE), which had a capacity of 5 L and was equipped with a magnetically coupled stirrer driving a paddle turbine. Specifically, 50 g of ground and defatted BSF larvae were suspended in 1 L of distilled water, and hydrolysis was conducted for 1 h and under stirring at 400 rpm.

Three hydrolysis tests were carried out: the first at a temperature of 160 °C (pressure 4.5 bar), the second at the same temperature of 160 °C and it was preceded by pretreatment with HCl (2N) for 24 h at room temperature and material to liquor ratio of 1/10 in a conical flask under stirring of 130 shakes/min. After filtration on wire mesh, neutralization with ammonia and rinsing with tap water, the solid part was hydrolyzed in an autoclave. The third test was conducted on grounded and defatted BSF larvae at a temperature of 180 °C corresponding to a pressure of 10 bar.

Each hydrolysate was filtered on a wire mesh, and the protein-rich liquid was further centrifuged at 8000 rpm for 15 min to remove precipitated solid material.

To determine the protein concentration, 10 mL from each liquid extract was dried in an oven at 105 °C for 4 h, and the dry weight was measured.

By measuring the final volume of each extract, the extraction yield was calculated using the formula:

(1) $$Extraction\;yield\;(\%)=\frac{Wf}{Wi}\times 100$$

where:

*wf*: dry weight of hydrolyzed proteins

*wi*: dry weight of defatted BSF larvae before hydrolysis

#### 2.2.3. Preparation of PVA/BSF Protein Blend Films

The protein hydrolysate from BSF larvae obtained from superheated hydrolysis at 160 °C for 1 h, having a protein concentration of 21 g/L, was placed in a flask in a thermostated bath at 65 °C for 1 h under stirring (130 shakes/min). PVA filament in water was treated under the same conditions and dissolved completely. The two solutions were mixed in the proportions PVA/BSF protein % *w*/*w* 100/0, 90/10, 70/30, 50/50, 30/70, 10/90 and 100/0 in a volume of 10 mL for each blend. The solutions at the different PVA/BSF protein concentrations were further mixed in a thermostatically controlled bath at 65 °C for 1 h under stirring (130 shaking/min) and then cast onto polyester plates and allowed to dry at room temperature for 48 h until complete water evaporation.

### 2.3. Characterization of PVA/BSF Protein Blend Films and BSF Protein Hydrolyzates

#### FT-IR Spectroscopy

BSF larvae, liquid and solid fractions after hydrolysis, PVA/BSF protein films and PVA/BSF films after biodegradation in soil were characterized using FT-IR spectroscopy in ATR (Attenuated Total Reflectance) mode with a diamond crystal. The Thermo Nexus spectrometer (Nicolet) instrumentation was used, and the spectra were processed with Omnic 9 software. Spectra were acquired with 64 scans in the wavenumber range 4000–650 cm^−1^ with resolution 4 cm^−1^ and gain 8.

### 2.4. Characterization of PVA/BSF Protein Blend Films

#### 2.4.1. Thermal Behaviour

Thermal characteristics were determined via Differential Scanning Calorimetry (DSC) and Thermogravimetric Analysis (TGA). A DSC 821e Mettler Toledo equipment (Schwerzenbach, Switzerland) was used, and data was processed using STARe SW 9.30 software. A 2–3 mg sample was inserted in an aluminium crucible and heated from 50 to 400 °C at a heating rate of 5 °C/min under a 10 mL/min nitrogen flow.

The relative crystallinity degree of PVA in the different films was calculated for reference to the pure PVA films by measuring the area of the melting peak corresponding to the melting enthalpy (*ΔH_m_*) through the formula:

(2) $$X_{c}(\%)=\frac{∆H_{m}}{\left(\frac{\%PVA}{100}\right)\times ∆H_{m,100\%PVA}}\times 100$$

where

*X_c_*: relative crystallinity degree of PVA in films at different PVA concentrations

*ΔH_m100%PVA_*: PVA melting peak area in pure PVA sample

*ΔH_m_*: PVA melting peak area in samples at different PVA concentrations

For TGA, thermograms were acquired using a TGA/DSC Mettler Toledo (Schwerzenbach, Switzerland) and processed using STARe SW 9.30. The software allows us to obtain both TGA curves and DTG derivatives. The analyses were carried out in 5–10 mg samples over a temperature range from 30 °C to 700 °C, with a constant heating rate of 10 °C/min under 70 mL/min nitrogen flow.

#### 2.4.2. Solubility in Water

Films were dried in an oven at 50 °C until constant weight and then immersed in 50 mL distilled water at room temperature for 24 h under shaking (130 shaking/min). The films were then filtered under a vacuum to recover solid parts and dried at 50 °C until constant weight, as reported by Gontard et al. [43]. Each experiment was repeated in triplicate.

Solubility in water was calculated using the formula:

(3) $$Solubility\;in\;water\left(\%\right)=\frac{w_{i}-w_{f}}{w_{i}}*100$$

where:

*w_i_*: initial sample dry weight

*w_f_*: final sample dry weight

#### 2.4.3. Water Uptake

PVA/BSF protein films were dried in an oven at 50 °C and then conditioned in a room at 20 °C and 65% of relative humidity for at least 24 h before weighing each sample every hour for 8 h and then after 24 h, as reported by Patrucco et al. [44].

Experiments were conducted in triplicate and water uptake was calculated using the formula:

(4) $$Water\;Uptake\left(\%\;w/w\right)=\frac{W_{f}-W_{i}}{W_{i}}*100$$

where:

*W_i_:* initial dry weight

*W_f_*: weight after *n hours in a conditioned environment

*n: 1, 2, 3, 4, 5, 6, 7, 8, 24 h

#### 2.4.4. Morphological Characterization

Scanning Electron Microscopy (SEM) analysis was carried out on EVO 10 (CarlZeiss AG, Oberkochen, Germany) equipment using SmartSEM software with 20 µA of the current probe, an acceleration voltage of 20 kV, and 30 mm working distance. PVA/BSF protein films were mounted on aluminium stubs and made to adhere using a double-sided adhesive tape. Before analysis, samples were sputter-coated with a thin gold layer under a rarefied argon atmosphere.

#### 2.4.5. Tensile Behaviour

Films were cut into strips 2 mm in width × 25 mm in length and were tested with a dynamometer ZwickRoell (GmbH Z005 ProLine 5 KN, Ulm, Germany) in a conditioned atmosphere at a constant rate of 10 mm/min according to the EN-ISO 5079 standard [45]. Data were processed using TestXPer III software. At least three measurements were carried out for each sample and tensile strength and elongation at break were reported. The thickness of each film was measured using a digital comparator (Fowler).

#### 2.4.6. Contact Angle

For the contact angle measurement, an optical goniometer EasyDrop model from Krüss Scientific GmbH (Hamburg, Germany) was used with the sessile drop method. A 10.5 μL milliQ water drop was deposited on the film surface with a syringe and the angle formed by each film surface and the drop profile was measured. A total of 8 measurements were performed on each film to obtain the mean and standard deviation.

### 2.5. Biodegradation Testing in Soil

PVA/hydrolyzed BSF protein films were dried at 50 °C, put in nylon mesh bags and buried under natural soil in a pot as reported by Bhavsar et al. [41]. The soil was kept moist by periodic irrigation, and samples were removed after 10/20/30/60/90 days and dried again at 50 °C.

The determination of biodegradability in soil was calculated using the following formula:

(5) $$Weight\;loss\left[\%\;w/w\right]=\frac{W_{i}-W_{f}}{W_{i}}\times 100$$

where:

*W_i_*: initial dry weight

W_f_: dry weight after *n dyes of burial in soil

*n: 10/20/30/60/90 days

FTIR spectra were acquired on each film after soil removal.

## 3. Results

### 3.1. Characterization of BSF Larvae and BSF Protein Extracts

The extraction hydrolysis yields referred to the dry weight of the defatted BSF larvae were 45.51% *w*/*w* for the trial carried out at 160 °C, 13.77% *w*/*w* for the trial at 160 °C after pretreatment with HCl, and 45.96% *w*/*w* for the trial at 180 °C. The time was always 1 h and the solvent was pressurized water, which offers the advantages of sterilizing the material and obtaining the extracts directly in water, avoiding costly and time-consuming purification processes.

The low yield of the process preceded by HCl pretreatment is presumably due to the loss of material during pretreatment, neutralization, and rinses, while the similar extraction yield of the 160 °C and 180 °C processes suggests that the temperature of 160 °C for 1 h is sufficient to extract the proteins and is not necessary to increase temperature and consume more energy.

**Figure 2:**
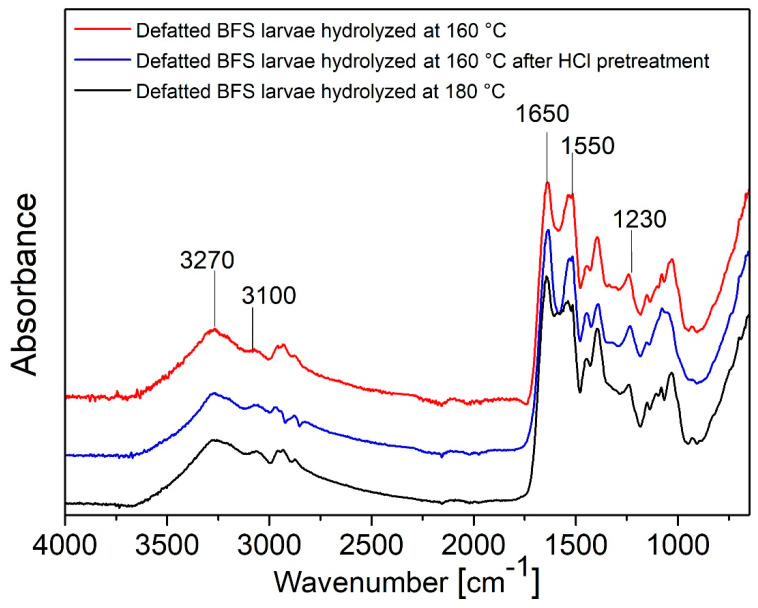
FTIR spectra of dried protein extracted from defatted BSF larvae at 160 °C, 160 °C after HCl pretreatment and 180 °C.

The FT-IR spectra of the three extracts are shown in Figure 2. They have similar absorptions mainly attributable to the presence of proteins. In fact, the peaks of Amide I at about 1650 cm^−1^ (C=O stretching), Amide II at about 1550 cm^−1^ (C=N stretching and N-H bending) and Amide III at about 1230 cm^−1^ (C-N stretching and N-H bending) are present and the peaks of Amide A at 3270 cm^−1^ and Amide b at 2930 cm^−1^ representing N-H stretching and C-H stretching, respectively, are also visible [46].

**Figure 3:**
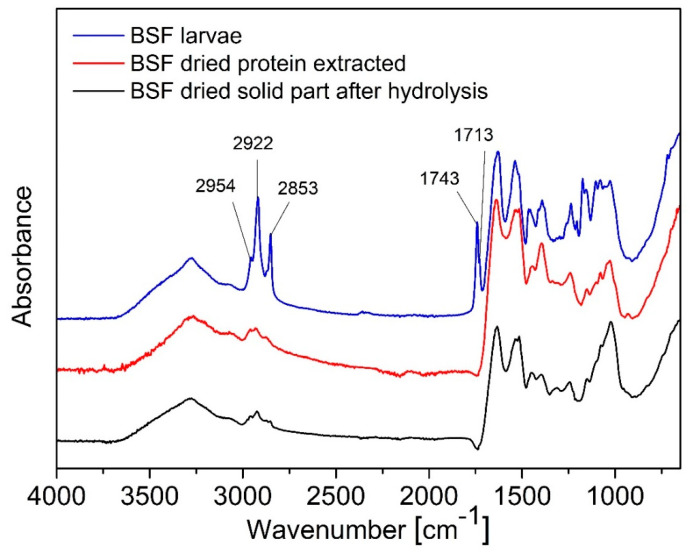
FTIR spectra of BSF larvae, BSF dried proteins extracted, BSF dried solid part after hydrolysis.

As observed in Figure 3, in BSF larvae spectra, there are present peaks from lipids at 2954 cm^−1^ (-CH_3_ asymmetrical stretch), at 2922 and 2853 cm^−1^ (symmetrical and asymmetrical stretching of -CH_2_), at 1743 cm^−1^ (-C═O stretch), and at 1713 cm^−1^ corresponding to carbonyl absorption [47]. All these absorptions are not present in the hydrolysate as grease was removed by extraction with hexane.

Moreover, in the solid part remaining as a deposit not extracted with the superheated water, there is a strong absorption mainly due to the mineral fraction with the characteristic peak of calcite (CaCO_3_) at 1030 cm^−1^ imputable to symmetric CO_3_ stretching [48] while the absorptions of the chitin present in the larvae overlap with non-solubilized protein and the inorganic parts absorptions [24]. Finally, as previously stated, protein peaks referable to amides are clearly visible in the dried extract.

### 3.2. Characterization of PVA/BSF Protein Blend Films

All PVA/BSF protein blends showed good film-forming ability except for the higher amount of protein blends (10/90 and 0/100% *w*/*w*), which produced brittle films hard to peel from the cast.

**Figure 4:**
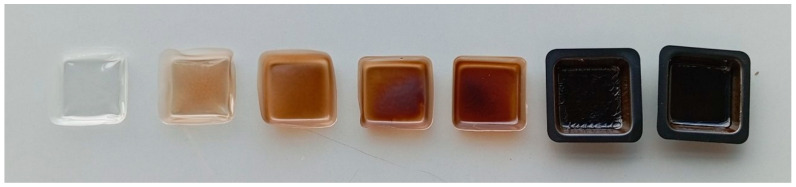
The visual appearance of PVA/BSF protein films in % *w*/*w* 100/0, 90/10, 70/30, 50/50, 30/70, 10/90 and 0/100 from left to right.

The films show visual properties influenced by their composition. They generally appear quite smooth with an even material distribution. However, in samples with a high concentration of protein, surface irregularities emerge. Regarding coloration, films with a higher percentage of PVA appear more transparent, while those with a high protein composition are more opaque and brownish in color (see Figure 4).

### 3.3. FT-IR Spectroscopy

**Figure 5:**
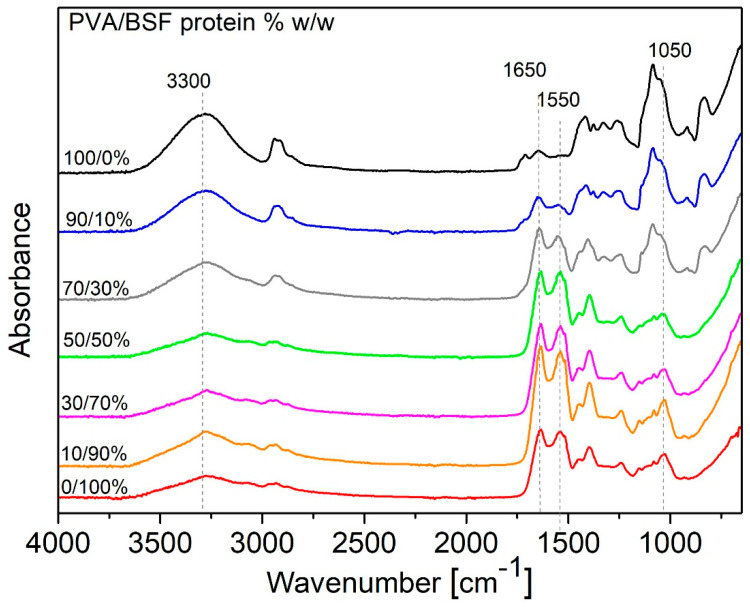
FTIR spectra of PVA/BSF protein films in % *w*/*w* 100/0, 90/10, 70/30, 50/50, 30/70, 10/90 and 0/100.

The PVA/BSF protein films at different compositions were characterized via FT-IR spectroscopy, as shown in Figure 5.

In films composed exclusively of PVA, the band located at about 3300 cm^−1^ appears broad and intense, highlighting intra- and intermolecular hydrogen bonds between the O-H groups of PVA. By increasing the protein concentration, this band broadens and reduces its intensity. Finally, in samples with low PVA amounts, the band becomes less defined, indicating the predominance of N-H stretching from BSF proteins.

In contrast, the Amide I band at about 1650 cm^−1^ and Amide II bands at about 1550 cm^−1^ are characteristic signals of proteins and increase with increasing BSF protein fraction in films [49].

Moreover, an intense peak at about 1100 cm^−1^ can be seen in the films with higher amounts of PVA, representing a symmetric C–C stretching mode or some stretching of the C–O bonds. This absorption is strongly associated with PVA crystallinity as referred to by Mansur et al. [50]. In blend films, this absorption reduces intensity as the protein content increases, showing a partial decrease in PVA content and its crystallinity.

From the FT-IR analysis, it can be concluded that PVA and BSF protein in films are bound by non-covalent bonds, and in particular, hydrogen bonds.

### 3.4. Thermal Behaviour

**Figure 6:**
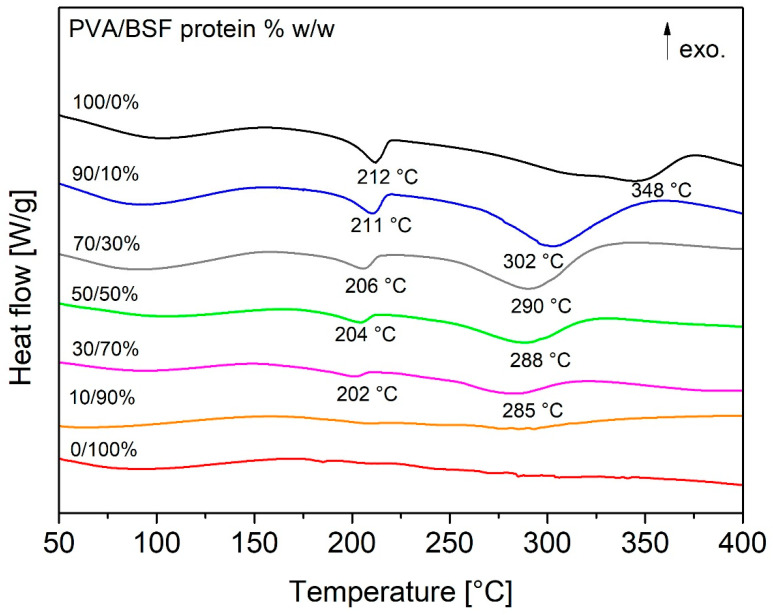
DSC traces of PVA/BSF protein films at different concentrations.

The DSC curves of the films at different percentages of PVA/BSF protein (see Figure 6) show a first endothermic peak of around 80 °C attributable to water evaporation and a second endothermic peak at around 212 °C for pure PVA, representing the melting point of the crystalline regions of PVA [51,52].

As the protein concentration increases in different films, there is a progressive decrease in the intensity of this peak correlated with the decrease in the crystallinity of PVA, while its temperature undergoes a progressive reduction from 212 °C of the pure PVA film to 201 °C of the PVA/BSF protein 30/70% *w*/*w* film. The percentage of crystallinity relative to the pure PVA sample of the different samples (except the PVA/BSF protein 10/90% *w*/*w* sample due to the absence of a significant crystalline phase) was calculated using Formula 2, and the results are shown in Table 1.

A progressive reduction in the relative crystallinity of PVA can be seen as the protein fraction increases, reaching the value of 30.68% in the 30% PVA sample. This trend can be attributed to interactions between PVA and BSF proteins, which insert between PVA polymer chains, limiting the PVA crystallization process, favoring a prevalence of amorphous regions within the films and affecting their physical and mechanical properties.

Finally, an additional endothermic event emerges in the DSC traces at higher temperatures that could be associated with protein degradation.

**Figure 7:**
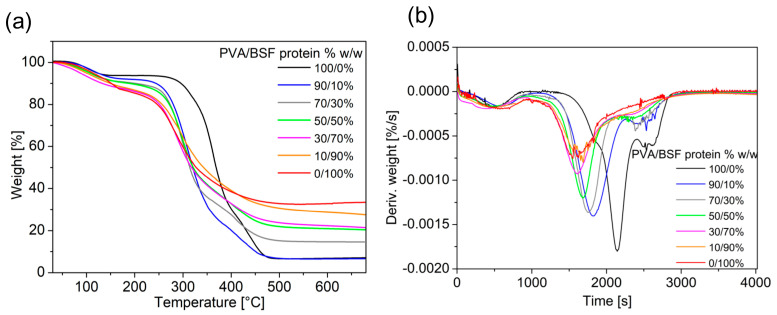
TGA traces of PVA/BSF protein films at different concentrations (**a**) and their first derivatives (**b**).

The results of thermogravimetric analysis of PVA/BSF protein films at different concentrations are shown in Figure 7 and summarized in Table 2.

TGA traces show an initial weight loss due to water evaporation at temperatures below 120 °C and degradation at a T_onset_ that increases with increasing PVA concentration. This behaviour indicates that PVA promotes a stabilizing effect on the polymer matrix, delaying the T_onset_ of thermal decomposition. In particular, the pure protein film exhibits the lowest temperature of 128.1 °C, consistent with the nature of proteins, which tend to degrade due to denaturation of their structures and disruption of intermolecular bonds. Minimal addition of PVA leads to a significant increase in T_onset_, which reaches 235.7 °C, highlighting the strong stabilizing impact of PVA. With increasing PVA concentration, a gradual T_onset_ increase is observed until it reaches 332.9 °C in the case of pure PVA.

The final residue shown in Table 2 was calculated as a function of the dry weight of the films, excluding the part of the curve where the weight loss is due to water evaporation.

The final residue of 35.5% *w*/*w* in the pure protein sample indicates the BSF protein’s tendency to leave a significant carbonaceous residue after thermal decomposition rather than decomposing completely into volatile compounds. The residue progressively decreases with the addition of PVA, showing that pyrolysis is increasingly complete due to the PVA contribution. A final residue of 7.6% *w*/*w* is achieved in pure PVA samples.

Finally, from the DTG (Derivative Thermogravimetry) curves, the peak temperature represents when the material decomposes at the maximum rate. In the pure protein sample, the DTG peak occurs at 280.8 °C, corresponding to the degradation of polypeptide chains [53]. As PVA increases in films, an increasingly pronounced shift of the peak toward higher temperatures is observed until it reaches 360.4 °C with pure PVA, confirming the stabilizing PVA impact.

### 3.5. Solubility in Water

**Figure 8:**
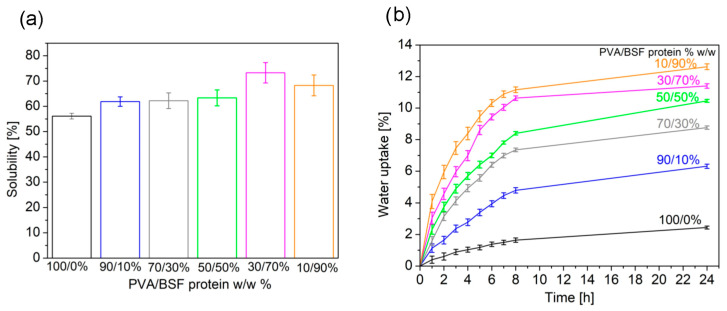
Solubility in water (**a**) and water uptake (**b**) of PVA/BSF protein films at different concentrations.

Experimental results (see Figure 8a) show different film solubilities in water depending on the concentration of PVA and protein. In general, as the concentration of PVA increases, a progressive decrease in film solubility is observed, and a decrease in the standard deviation related to inhomogeneity in film structure. This behaviour reflects both the higher solubility of BSF protein compared to PVA and a structural transition of the material from an amorphous to a more crystalline configuration that is less accessible to water [54]. The decrease in the PVA crystallinity correlated with the increase in protein concentration in films was demonstrated via previous DSC analysis.

### 3.6. Water Uptake

Analysis of water uptake curves (see Figure 8b) shows a significant increase in water uptake in the first eight hours for all the PVA/BSF protein films analyzed, followed by a gradual slowdown leading to a plateau within 24 h.

Films with a high concentration of PVA show consistently lower water uptake values at each time step. This behavior is attributable to the crystalline structure of PVA, which organizes its polymer chains into ordered and compact configurations, reducing the space available for water molecules and limiting the mobility of polymer chains, which further hinders water uptake. In addition, the low standard deviation observed in these samples suggests homogeneous and predictable behavior due to the uniform distribution of PVA within the material. In contrast, films with higher protein concentrations exhibit significantly higher water uptake. Proteins, predominantly amorphous, offer more hydrophilic sites, such as amine and carboxyl groups, which can form hydrogen bonds with water. This disordered structure promotes rapid absorption in the early stages and a greater ability to retain moisture. In addition, the data show greater variability in samples with high protein content, which can be attributed to local inhomogeneities in protein distribution.

### 3.7. Morphological Characterization

**Figure 9:**
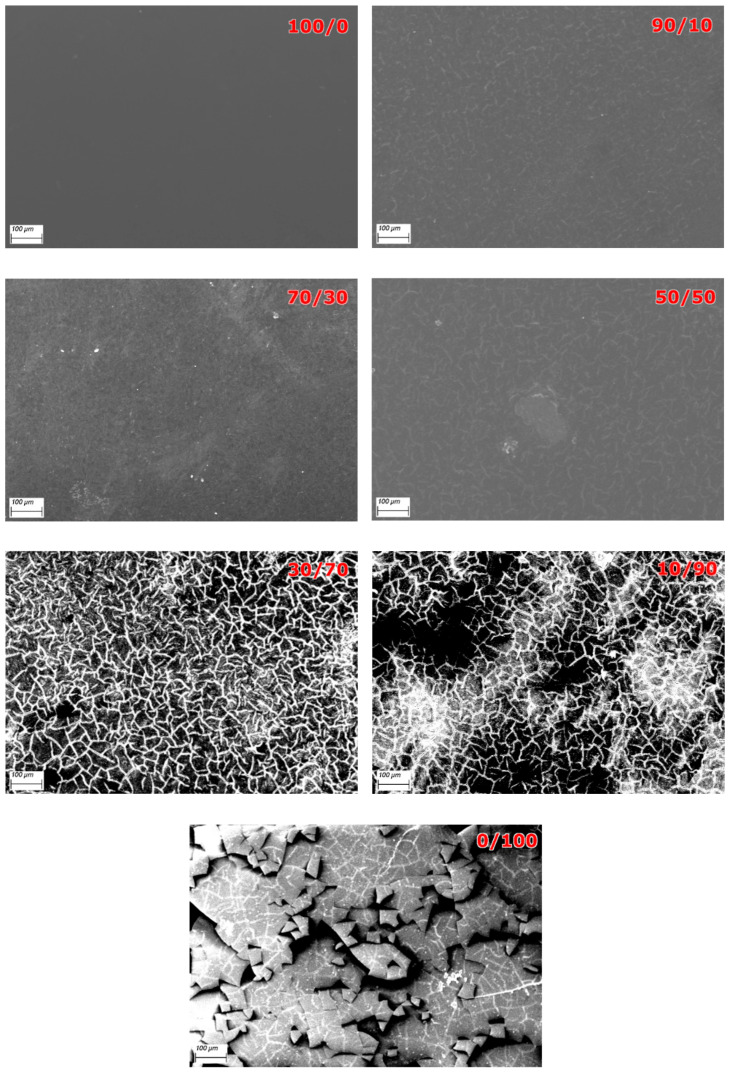
SEM pictures at 100× of PVA/BSF protein films at different concentration 100/0, 90/10, 70/30, 50/50, 30/70, 10/90 and 0/100 (% *w*/*w*).

Figure 9 shows the surfaces of the PVA/BSF protein films at different concentrations at 100× magnification.

For the pure PVA film, the surface appears smooth and homogeneous, confirming that the material forms an isotropic and stable matrix. At 10% protein addition, slight inhomogeneities emerge, but no porosity is detected and the polymer matrix retains its cohesion. Increasing the protein amount, small, randomly distributed cracks begin to appear, and some areas of the film appear darker, suggesting the presence of microvoids or areas of reduced density, given the onset of phase separation between PVA and BSF protein. In PVA/BSF 30/70% *w*/*w* protein films, the surface is characterized by a network of interconnected cracks, making the material less resistant to mechanical stress. In PVA/BSF 10/90% *w*/*w* protein films, proteins aggregate and don’t integrate properly with the remaining PVA. Moreover, film porosity is very pronounced, and the uneven distribution of proteins creates weak points that promote fracture formation. Finally, the neat protein film shows separate fragments. Analogous results were obtained by Rajabinejad et al. in similar materials [55].

### 3.8. Tensile Behaviour

The tensile mechanical properties of the films at different PVA/BSF protein concentrations are summarized in Table 3. For pure PVA films, the tensile strength of 8 MPa is attributable to the uniform and stable crystal structure of PVA, which ensures a homogeneous distribution of mechanical stress. By introducing protein, the tensile strength first slowly increases until 70/30% *w*/*w* PVA/BSF protein, suggesting that protein acts as a plasticizer, promoting a balanced stress distribution and stability of the crystalline network. When increasing the amount of protein until 70% *w*/*w*, however, the tensile strength decreases strongly as the crystalline network appears to be compromised by protein dominance. As the amount of protein increases, the elongation at break gradually increases until 30% *w*/*w* of protein. In particular, for the neat PVA film, the elongation at the break is 160.6%, which reflects a medium ductility of this polymer. Adding 10% and 30% protein, the film strain rises slightly, suggesting an increase in plasticization. As the protein content increases, the strain decreases and the material becomes brittle. At 50% and 30% protein content in the films, little necking has been observed. 

### 3.9. Contact Angle

From Table 4, it can be seen that as the percentage of protein increases, the contact angle decreases from 67.6 ± 0.41° of pure PVA to 53.7 ± 0.95° of PVA/BSF protein 30/70% *w*/*w*, indicating greater film hydrophilicity. In addition, the standard deviation becomes more and more pronounced due to the increasingly irregular surface. Pure PVA film has hydrophilic functional groups; however, in the crystalline component, the denser packing of polymer chains limits exposure of hydrophilic groups, which has a direct impact on the wettability properties of the material. In films with 90% PVA, the proteins introduce polar groups that promote hydrogen bonds with water. Although PVA remains predominant, it maintains some resistance to liquid diffusion. As protein content increases, the contact angle decreases further due to protein functional groups facilitating interaction with water molecules. In addition, the increase in hydrophilicity as the protein amount increases could be related to the roughness of the films, as shown by SEM images [56].

### 3.10. Biodegradation Testing in Soil

**Figure 10:**
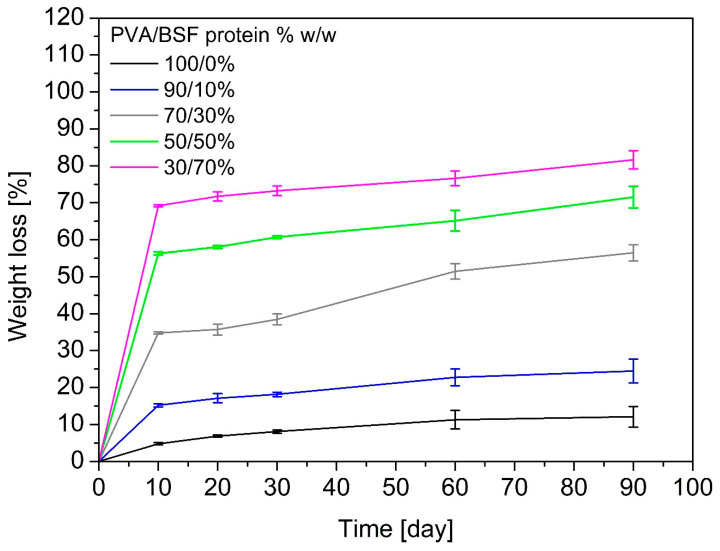
Weight loss of PVA/BSF protein films with different compositions after 10, 20, 30, 60, and 90 days of burial in the soil.

Figure 10 summarizes the weight losses of PVA/BSF protein films at different concentrations after 10, 20, 30, 60 and 90 days of burial in soil.

The curves show that samples with a higher protein content degrade more rapidly, while films with a higher percentage of PVA are more resistant to soil degradation. The degradation is generally characterized by a rapid initial mass loss, followed by a gradual slowdown. The pure PVA film shows a weight loss of 12% *w*/*w* after 90 days, confirming poor biodegradability in the soil, as confirmed by other authors [57], because few microorganisms present in the soil can biodegrade PVA [58]. As the percentage of protein increases, the sample weight loss increases until 80% *w*/*w* after 90 days of soil burial for the 30/70% *w*/*w* PVA/BSF protein sample. Lowering and disappearance of the Amide I (1650 cm^−1^) and Amide II (1550 cm^−1^) absorption peaks in FTIR spectra of biodegraded films highlight that the proteins are degraded in soil faster than PVA. This behaviour of rapid protein degradation in soil is confirmed by Dani et al. [59].

## 4. Conclusions

The present work focused on the production and characterization of bioplastics, combining PVA with proteins extracted from BSF larvae with superheated water without the use of harsh chemical reagents. The main objective was to develop a material which is adaptable to different applications, sustainable and biodegradable, and which can be a viable alternative to conventional plastics, which are not biodegradable and obtained from non-renewable sources. The experimental approach has started from the preparation of raw materials until the characterization of the properties of bioplastics obtained from PVA/BSF protein at different concentrations. FTIR analysis showed the formation of hydrogen bonds that ensure good cohesion of the polymer matrix. However, a nonuniform distribution of proteins within the polymer matrix is shown, with a tendency for aggregate formation affecting the material’s mechanical properties, with decreased tensile properties in films with high protein content. In addition, films with higher protein content show higher water uptake, water solubility, hydrophilicity, and faster biodegradation in soil than films with higher PVA content. PVA/BSF protein bioplastics can be used as biodegradable packaging or as agricultural mulches, modulating the relative amount of PVA and BSF protein and taking advantage of the increased mechanical strength of films with higher concentrations of PVA and the increased biodegradability of films with a higher content of BSF protein. The optimization of these materials and their introduction into large-scale production processes could represent important progress for the sustainable materials industry, helping to reduce the environmental impact associated with the use of traditional plastics.

## Figures and Tables

**Table 1:** PVA relative crystallinity degree and melting enthalpy area for its calculation.

PVA/BSF Protein (% *w*/*w*)	ΔH_f_ [J/g]	*X_c_* [%]
100/0	−52.73 ± 0.24	100
90/10	−43.385 ± 2.42	82.28 ± 6.48
70/30	−34.01 ± 1.46	64.50 ± 3.92
50/50	−22.25 ± 1.09	42.20 ± 2.92
30/70	−16.18 ± 0.83	30.68 ± 2.23

**Table 2:** TGA parameters of PVA/BSF protein films at different concentrations.

PVA/BSF Protein [% *w*/*w*]	T_onset_ [°C]	T_peak_ [°C]	Residue [% *w*/*w*]
100/0	332.9 ± 0.8	360.4 ± 2.9	7.6 ± 0.3
90/10	274.6 ± 1.8	302.5 ± 10.7	7.7 ± 1.1
70/30	267.7 ± 0.6	294.7 ± 6.2	15.1 ± 0.5
50/50	259.3 ± 1.5	291.8 ± 10.7	24.2 ± 3.7
30/70	241.8 ± 1.2	284.6 ± 7.2	23.3 ± 0.8
10/90	235.7 ± 0.7	283.7 ± 3.5	29.4 ± 2.3
0/100	128.1 ± 1.5	280.8 ± 2.6	35.5 ± 1.6

**Table 3:** Tensile strength, elongation at break and modulus of PVA/BSF protein films at different concentration.

PVA/BSF Protein [% *w*/*w*]	Tensile Strength [MPa]	Elongation at Break [%]
100/0	8 ± 1.04	160.6 ± 14.0
90/10	11.3 ± 2.55	236.4 ± 38.7
70/30	11.35 ± 0.68	212.8 ± 40.4
50/50	6.85 ± 1.05	43.75 ± 4.45
30/70	3.21 ± 0.84	26.6 ± 16.8

**Table 4:** Contact angle values of PVA/BSF protein films at different concentrations.

PVA/BSF Protein [% *w*/*w*]	Contact Angle
100/0	67.6 ± 0.41°
90/10	65.6 ± 0.54°
70/30	59.7 ± 0.67°
50/50	56.1 ± 0.80°
30/70	53.7 ± 0.95°

## Data Availability

All the data will be made available on specific request.
